# Prevalence of glutamine deficiency in ICU patients: a cross-sectional analytical study

**DOI:** 10.1186/s12937-016-0188-3

**Published:** 2016-08-02

**Authors:** Arista Nienaber, Robin Claire Dolman, Averalda Eldorine van Graan, Renee Blaauw

**Affiliations:** 1 Centre of Excellence for Nutrition, North-West University, Potchefstroom Campus, Potchefstroom, South Africa; 2 Division of Human Nutrition, Faculty of Medicine and Health Sciences, Stellenbosch University, PO Box 241, Cape Town, South Africa

**Keywords:** Glutamine, Intensive care unit, C-reactive protein, Interleukin-6, Gender

## Abstract

**Background:**

Not only is glutamine deficiency an independent predictor of mortality in intensive care unit (ICU) patients, but glutamine supplementation is also recommended for its proven outcome benefits. However, recent data suggest that early glutamine supplementation in certain patient groups increase mortality. The aim of this study was to investigate plasma glutamine levels of adult ICU patients in the South African setting and to determine relationships between glutamine levels, gender, diagnostic categories and selected inflammatory markers. The data from this study will be used as baseline measurement to support a large scale study that will be undertaken in the South African ICU population.

**Methods:**

This cross-sectional, analytical study included 60 mixed adult ICU patients within 24 h post ICU admission. Plasma glutamine levels were determined on admission. The relationship between glutamine levels, Interleukin-6 (IL-6) and C-reactive protein (CRP); as well as gender- and diagnosis-related differences in glutamine levels were also investigated. A non-parametric ROC curve was computed to determine the CRP concentration cut-off point above which glutamine becomes deficient.

**Results:**

The median plasma glutamine level (497 μmol/L) was in the normal range; however, 38.3 % (*n* = 23) of patients had deficient (<420 μmol/L) and 6.7 % (*n* = 4) had supra-normal glutamine levels (>930 μmol/L). No significant difference could be detected between glutamine levels and gender or diagnosis categories as a group. When only the medical and surgical categories were compared, the median plasma glutamine level of the medical patients were significantly lower than that of the surgical patients (*p* = 0.042). Glutamine showed inverse associations with CRP levels (*r* = −0.44, *p* < 0.05) and IL-6 concentrations (*r* = −0.23, *p* = 0.08). A CRP cut-off value of 95.5 mg/L was determined above which glutamine levels became deficient.

**Conclusions:**

About a third of patients (38 %) were glutamine deficient on admission to ICU, whereas some presented with supra-normal levels. While glutamine levels correlated inversely with inflammatory markers, and a CRP value of above 95.5 mg/L indicated potential glutamine deficiency, the clinical application of this finding needs further investigation.

## Background

Pharmaconutrition refers to nutrients that are administered as pharmacological agents, forming part of the medical treatment plan. It is currently applied in various clinical settings to improve patient outcomes [1]. Glutamine, the most abundant non-essential amino acid, is the most-researched pharmaconutrient to date [2]. Previous studies showed decreased levels of plasma and muscle glutamine in selected critically ill, post-surgical, multiple trauma, burns, septic and general intensive care unit (ICU) patients [3–5], resulting in the classification of glutamine as a “conditionally essential amino acid” under certain circumstances. Glutamine deficiency and, therefore, its unavailability to perform important functions contributes to an inappropriate response to stress and injury, ultimately leading to an increased ICU and post-ICU mortality risk.

Glutamine supplementation has been extensively studied for its contribution to the improvement of patient outcomes. Until recently, it has been deemed safe and effective in a variety of patient groups, including the severely ill, burns and surgical patient [6–9]. Outcome benefits such as reductions in length of hospital stay (LOHS), length of ICU stay, mortality risk and infectious complications, as well as an improvement in nitrogen balance, was previously reported, depending on the specific patient group as well as the dose and route of administration [6–9].

The Reducing Deaths due to Oxidative Stress (REDOXS) study questioned the safety and applicability of glutamine supplementation in all patient groups. In this study an increased mortality risk was found when supplementing multiple organ failure (MOF) patients with high dosages of enteral and parenteral glutamine within 24 h after ICU admission [10]. In a sub-sample of this study only 31 % of patients were glutamine deficient (<420 μmol/L) while 15 % had supra-normal levels (>930 μmol/L). Moreover, Rodas et al. [11] found that a U-shaped curve represents the association between glutamine and mortality, where both low (<420 μmol/L) and high (>930 μmol/L) levels were associated with a higher mortality risk.

It is, therefore, important to determine the glutamine status of patients in order to determine those requiring supplementation and those where supplementation may cause harm. In order to do this, it would be ideal to measure plasma glutamine levels of all patients prior to supplementation, but it is not routine practice in most clinical settings. Furthermore, no South African (SA) based data are available on plasma glutamine levels in the ICU setting. Consequently, identifying markers, which could aid in detecting patients in need of glutamine supplementation or those where glutamine is contraindicated, has become a priority. The main aim of this study, was to examine plasma glutamine levels of SA adult ICU patients on day one of admission. In addition, the influences of gender and different diagnostic categories on glutamine status were investigated. Previously a relationship between inflammation and glutamine levels has been reported. More specifically, an inverse association between interleukin-6 (IL-6) and glutamine were established [12, 13]. This is also the case for the inflammatory marker C-reactive protein (CRP) and amino acids [14]. Therefore, the relationship between glutamine levels and selected inflammatory markers, CRP and IL-6, were investigated to confirm earlier findings on IL-6 and to determine whether a relationship with CRP exist. The findings of this research study will be used as baseline data for a larger SA based study conducted in 2016.

## Methods

### Study design and setting

This observational cross-sectional study was performed at two ICU settings in the North West province, SA. The units are both mixed ICUs, consisting predominantly of medical patients, but also admitting surgery and trauma patients. The study was carried out in accordance with the declaration of Helsinki and ethical approval was obtained from the North-West University Human Research Ethics Committee (NWU-00186-13-S1); the Policy, planning, research monitoring and evaluation committee of the North West Department of Health; and the Patient Safety Groups of both hospitals. Informed, voluntary consent to participate in the study was obtained from all participants or their legal proxy.

### Patients

All adult patients (>18 years) admitted to the ICUs of the two participating hospitals during the study period (March to August, 2014) were eligible for inclusion, provided they met the following criteria: informed consent obtained and blood sampling conducted within 24 h post-ICU admission. Patients excluded were those receiving total parenteral nutrition with added glutamine prior to blood sampling; those receiving glutamine-enriched enteral feeding, high protein or amino acid-based oral supplements prior to blood sampling; and those who were pregnant or lactating. From the admission records of the hospital, a dropout analysis was performed to identify the characteristics of the patients admitted to the ICU during the study period, but not included in the study.

### Blood sampling and laboratory analysis

Venous blood samples were taken within 24 h following ICU admission. Plasma samples for glutamine and IL-6 analysis were collected in ethylene diamine tetra-acetic acid (EDTA) tubes and centrifuged within 30 min at 3000 × g for 15 min (4 °C). Plasma was then stored at –80 °C within one hour post-sampling and for no longer than 30 days, pending analysis. Plasma glutamine levels were determined by an adapted method of the EZ:faast amino acid analysis procedure (Phenomenex, Torrance, CA, USA) for the analysis of free physiological amino acids, using gas chromatography–mass spectrometry (Hewlett Packard HP 6890 series Gas Chromatography system, Palo Alto, CA, USA and Agilent 5973 N Mass Selective Detector, Santa Clara, CA, USA). Plasma glutamine levels of below 420 μmol/L were classified as deficient and above 930 μmol/L as supra-normal [11, 15].

IL-6 was measured by means of the IL-6 Quantikine enzyme-linked immunosorbent assay (ELISA) kit (R&D systems, Minneapolis, MN, USA), using the Thermofisher Scientific Multiskan FC (Vanta, Finland). For the purpose of this research, IL-6 levels below 10 pg/mL were regarded as normal [16].

For the determination of CRP concentrations, serum gel tubes were prepared for analysis by centrifuging samples at 4000 rpm for 20 min. Analysis was conducted by means of the Sequential Multiple Analyser Computer, using the Beckman Coulter DxC880i (Beckman Coulter, Ireland). A reference CRP concentration of below 5 mg/L was regarded as normal, whereas mild inflammation, active and severe infection were classified according to 10–49 mg/L, 50–200 mg/L and >200 mg/L respectively [17, 18].

### Statistical analysis

The computer software package IBM SPSS® Statistics 22 (Statistical Package for Social Sciences, IBM, NY, USA) was used for statistical analysis. A *p*-value of ≤0.05 was considered statistically significant. Non-parametric data were log-transformed to improve normality and are reported as geometric means and 95 % confidence interval (CI). Normally distributed data are reported as means (95 % CI), and data that are still non-parametric following log transformation, as medians [25^th^-75^th^percentiles]. A Mann–Whitney *U* test were used to compare the median plasma glutamine level difference between genders.

The influence of different diagnoses on plasma glutamine levels was examined by using the Kruskal-Wallis analysis of variance (ANOVA) with the Bonferroni adjustment. In order to determine the relationship between plasma glutamine levels and inflammatory markers, the Spearman rank test was used. Other variables that could influence the levels of plasma glutamine, were included in the statistical models using analysis of covariance (ANCOVA). A receiver operating characteristic (ROC) curve was computed to determine the CRP cut-off value at which glutamine becomes deficient.

## Results

During the study period 60 ICU patients were included in this study. The baseline characteristics of the patients are presented in Table 1.

**Table 1:** Baseline characteristics of the ICU patient group

Characteristic	ICU patients	
Age, year^a^	46 (32.3; 57.8)	
	*n*	%
Gender:		
Female	25	41.7
Male	35	58.3
ICU admission category:		
Medical	43	71.7
Surgery^b^	15	25.0
Trauma	2	3.3
Reason for ICU admission:		
Cardiovascular/vascular	10	16.7
DKA/HONK	13	21.7
Gastrointestinal disorder	5	8.3
Liver failure	1	1.7
Neurological disorder	2	3.3
Post-surgery	9	15.0
Respiratory disorder	9	15.0
Renal failure	2	3.3
Septic shock	3	5.0
Trauma	2	3.3
Other	4	6.7

*DKA* diabetic ketoacidosis, *HONK* hyperosmolar non-ketotic coma, *ICU* intensive care unit, *n* number/ frequency

^a^Age reported as mean (95 % CI)

^b^Surgery patients include: neurosurgery (*n* = 3), elective surgery (*n* = 4) and emergency surgery (*n* = 8) patients

In Table 2, the median plasma glutamine levels of different medical condition categories are presented, together with the distribution of patients among deficient (<420 μmol/L), normal (420-930 μmol/L), or supra-normal (>930 μmol/L) levels in each category.

**Table 2:** Glutamine status of the study population

Group	Median plasma GLN level (μmol/L) medians [25^th^ – 75^th^ percentiles]	*P*-value	Deficient: <420 μmol/L	Normal: 420 – 930 μmol/L	Supra-normal: >930 μmol/L
			*n* (%)		
ICU patients (*n* = 60)	497 [387 – 644]		23 (38.3)	33 (55.0)	4 (6.70)
ICU men (*n* = 35)	558 [395 – 697]	0.116	12 (34.3)	19 (54.3)	4 (11.4)
ICU women (*n* = 25)	466 [380 – 543]		11 (44.0)	14 (56.0)	–
Admission categories:					
Medical (*n* = 42)	475 [372 – 627]	0.325**	18 (42.8)	23 (54.8)	1 (2.40)
Surgical (*n* = 16)	515 [468 – 782]		4 (25.0)	9 (56.2)	3 (18.8)
Trauma (*n* = 2)	432 [381 – 432]		1 (50.0)	1 (50.0)	–
Specific conditions:^b^					
HIV+ (*n* = 8)	562 [375 – 1062]		2 (25.0)	5 (62.5)	1 (12.5)
Liver failure (*n* = 4)	497 [389 – 643]		2 (50.0)	1 (25.0)	1 (25.0)
Renal failure^a^ (*n* = 7)	575 [388 – 714]		3 (42.9)	4 (57.1)	–
MOF (*n =* 4)	355 [310 – 689]		3 (75.0)	1 (25.0)	–
Sepsis (*n* = 4)	355 [310 – 689]		3 (75.0)	1 (25.0)	–
DM (*n* = 19)	380 [273 – 500]		10 (52.6)	9 (47.4)	–

Non-parametric data reported as median [25^th^–75^th^percentile]

*DM* diabetes mellitus, *GLN* glutamine, *HIV+* human immunodeficiency virus seroreactive, *ICU* intensive care unit, *MOF* multiple-organ failure, *n* number/ frequency

^a^Patients not dialysed; ^b^Median plasma glutamine levels and distribution for certain conditions, that are expected to alter glutamine status

**p-value 0.042 when excluding trauma patients and comparing the medical and surgical patients

Of the patients 38.3 % were glutamine deficient, while 6.7 % had supra-normal levels. A non-significant difference was found comparing the median plasma glutamine levels of female and male patients (*p* = 0.116), and between the different diagnostic categories (medical, surgery, trauma) (Table 2). The inability to demonstrate a difference between the groups was possibly related to the statistical power, as very few trauma patients were admitted to the ICUs during the study period. However when excluding the two trauma patients, a significantly lower median glutamine level was found in the medical patient group compared to the surgical group (*p* = 0.042) (after adjusting for age). Of the 42 medical patients included, 42.8 % had deficient plasma glutamine levels, whereas this was true for only 25 % of the surgical group (Table 2). One patient in the medical group and three of the patients admitted for surgical reasons had supra-normal plasma glutamine levels. Table 2 elaborates on these conditions.

**Fig. 1:**
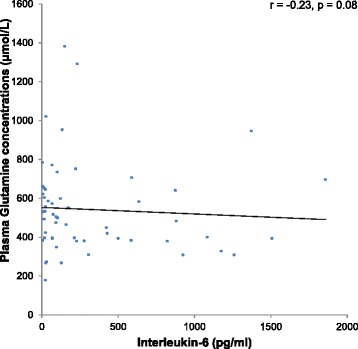
Relationship between plasma glutamine and interleukin-6 levels (*r* = −0.23, *p* = 0.08)

**Fig. 2:**
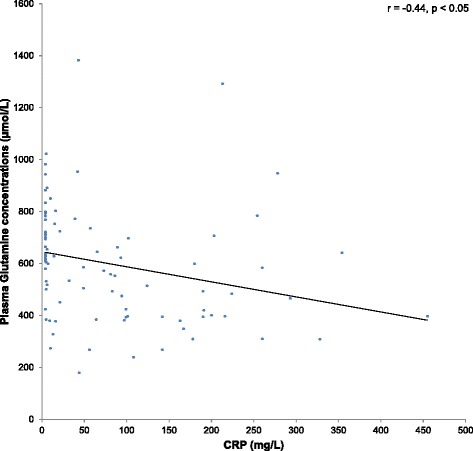
Relationship between plasma glutamine and CRP levels (*r* = −0.44, *p* < 0.05). CRP, C-reactive protein

Additionally, the association between plasma glutamine levels and inflammatory markers (CRP and/or IL-6 levels) was investigated. A trend towards an inverse association could be found between plasma glutamine levels and IL-6 (*r* = −0.23, *p* = 0.08) (Fig. 1). After adjusting for age by means of a partial correlation, the correlation remained insignificant. However, a significant inverse association was demonstrated between CRP and plasma glutamine levels (*r* = −0.44, *p* < 0.05) (Fig. 2).

**Fig. 3:**
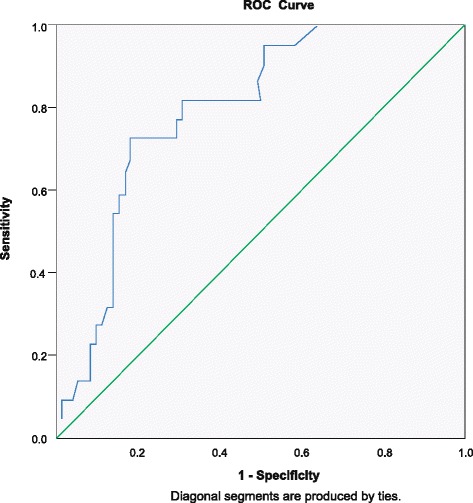
The receiver operating characteristic curve, computing the C-reactive protein level above which glutamine becomes deficient. Area under curve (AUC): 0.759 (95 % CI 0.653; 0.865, *p* < 0.0001)

A non-parametric ROC curve was computed to determine the CRP level cut-off point at which plasma glutamine levels became deficient (Fig. 3). The optimal cut-off was obtained from the Youden index value at 95.5 mg/L [Sp (Specificity) = 69 %, Sn (Sensitivity) = 62.2 %, AUC (area under the curve): 0.759 (95 % CI 0.653; 0.865), *p* < 0.0001]. Of the patients that had CRP values above 95.5 mg/L (*n* = 25), 74 % had plasma glutamine levels below 420 μmol/L.

**Fig. 4:**
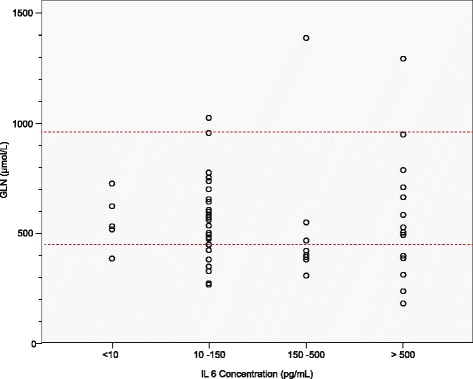
Plasma glutamine levels presented per interleukin-6 category. The area between the dashed lines represents normal plasma glutamine reference ranges. GLN, glutamine; IL-6, interleukin-6

**Fig. 5:**
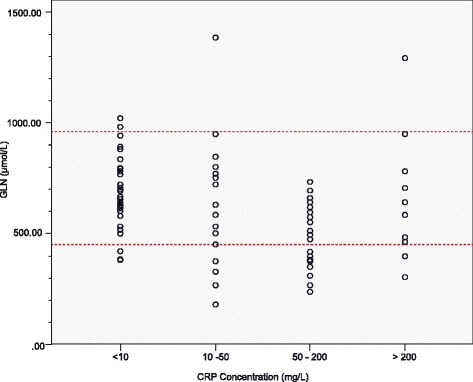
Plasma glutamine levels presented per C-reactive protein category. The area between the dashed lines represents normal plasma glutamine reference ranges. GLN, glutamine; CRP, C-reactive protein

The IL-6 and CRP values were also categorised to present the plasma glutamine levels visually in different categories of inflammation (Figs. 4 and 5). It was evident that the majority of patients with normal IL-6 levels (<10 pg/mL), i.e., without inflammation, also had normal plasma glutamine levels. On the other hand, those with inflammation presented with lower plasma glutamine levels, especially those in the 150–500 pg/mL IL-6 and 50–200 mg/L CRP groups. In the hyper-inflammatory groups (IL-6 > 500 pg/mL and CRP >200 mg/L) the glutamine levels were scattered, ranging from very high to very low plasma glutamine levels.

## Discussion

This study aimed to establish the plasma glutamine levels in a group of mixed ICU patients. It is important as the measurement of glutamine levels is currently not readily available, expensive and impractical in most hospital settings. This research serves as a pilot study for a larger study to be conducted in the SA setting, which will further elaborate on plasma glutamine levels throughout ICU stay, taking into account severity of illness as well as nutritional status of the patients. In order to obtain a representative sample of a real-life ICU population, all patients admitted to the chosen ICUs during the study period were eligible for inclusion.

The median plasma glutamine level of this ICU population (497 μmol/L [387–644 μmol/L]) was in the normal range, with 38.2 % categorised as glutamine deficient. This is comparable with findings by Oudemans-van Straaten et al. [15], indicating a median ICU admission level of 495 μmol/L (interquartile range 350–600 μmol/L) together with other research reporting that 31 to 43 % of ICU patients had deficient glutamine levels on day one of ICU admission [11, 15, 19].

The mechanism whereby glutamine becomes depleted is attributed to its translocation from the muscle to supply increased utilisation rates by vital splanchnic organs [20]. Finally, *de novo* synthesis may decrease and be insufficient to meet increased demands, resulting in glutamine depletion [20–22]. Low plasma glutamine levels are possibly life threatening, as they reflect the unavailability of glutamine to fulfil its important functions. In addition, deficient admission plasma glutamine levels are an independent predictor of increased ICU and post-ICU mortality rates [11, 15].

Supplementing patients who are not glutamine deficient may pose a risk, as Rodas et al. [11] reported a U-shaped curve association between plasma glutamine levels and mortality. In this study, only 6.7 % of patients had supra-normal plasma glutamine levels. Similarly, Heyland et al. [19] reported supra-normal admission levels in 15 % of MOF, ICU patients. Other studies also found high levels in selected patients [11, 15]. Both modest and very high plasma glutamine levels are attributed to organ failures, such as renal and acute liver failure [11, 23–25]. In this study group, however, only one of the acute liver failure patients had a glutamine level above 930 μmol/L. Nevertheless, the two other liver failure patients had chronic liver failure, which is thought to rather lead to glutamine deficiency.

When considering diagnostic categories, it was difficult to distinguish differences between the glutamine statuses. In a recent study it was found that 60 % of trauma ICU patients presented with deficient plasma glutamine levels prior to glutamine supplementation [5]. In the current study no conclusion could be drawn as to how trauma, as primary diagnosis, influenced glutamine levels, due to the few trauma patients included. Almost half of those admitted for medical reasons had deficient levels on ICU admission. They also had significantly lower glutamine levels than surgical patients. Diabetes, was previously thought not to influence glutamine status [26, 27]. Interestingly, however, when investigating the patients admitted for medical reasons in further detail, the majority of diabetic patients had deficient admission levels. On the other hand some of the included patients with renal-, liver, MOF and sepsis, as determined by clinical history, physical findings and blood cultures, had deficient and normal glutamine levels, contrary to previous reports of elevated levels to be expected in these patients [19, 28].

Earlier research suggested that a positive HIV status cause reduced plasma glutamine levels due to secondary infections and an increased need to maintain immune balance [29]. Findings in the current study showed that HIV reactive patients predominantly had normal glutamine levels. This could be ascribed to the fact that the inflammatory response of the HIV positive patients in this study were lower than expected, since there were no statistically significant difference between HIV positive and negative individuals with regard to either CRP (*p* = 0.271) or IL-6 levels (*p* = 0.654). It should be mentioned that HIV status was not tested, but only recorded if already diagnosed, and the disease progression was not documented. Nevertheless, this was not the typical glutamine profile that one would expect.

Regarding post-surgery patients, reduced plasma glutamine levels have been well established as a characteristic of their amino acid profile [3, 30, 31]. In the current study, more than half of the surgery patients presented with normal glutamine levels. However, the degree to which levels are affected may be related to the severity, complexity, and duration of the surgery, which was not within the scope of this study [31]. These findings stress the difficulty to make assumptions on glutamine status based only on the patient’s diagnosis.

Other factors that may have influenced plasma glutamine levels were also considered. In earlier studies, plasma glutamine levels were found to be significantly associated with gender in healthy individuals [32]. However in this study no significant gender-related difference could be detected in the glutamine levels of patients. It confirms findings by Viggiano et al. [31], who similarly reported gender not to influence glutamine levels post-operatively.

The current research further indicates, in support of previous findings, that a definite relationship between plasma glutamine and inflammation exists. Two well-known inflammatory markers, IL-6 and CRP, were measured. IL-6, described as an early marker of inflammation, drives inflammation, causing the release of CRP and other markers of the acute-phase response. It is also a predictor of the severity of the injury and major complications [33]. A trend towards an inverse association was found between plasma glutamine and IL-6 levels. This is supported by earlier studies reporting a negative association between IL-6 and glutamine [12, 13].

CRP is an inflammatory marker frequently measured in the hospital setting. Suliman et al. [14] reported an inverse association between CRP and amino acids. Parenteral glutamine supplementation has further been found to significantly reduce CRP concentrations [34, 35]. In the current study an inverse association was found between CRP and glutamine levels (*p* < 0.05). A cut-off point of 95.5 mg/L was determined as the CRP value above which glutamine levels can potentially be expected to become deficient. This is a novel finding and can possibly be used in the identification of patients at risk for glutamine deficiency. When interpreting the plasma glutamine levels grouped per category of CRP values, it is clear that the lower glutamine levels are evident in patients with CRP levels that fall in the 50 to 200 mg/L CRP value group (Fig. 5). However, those with CRP values above 200 mg/L presented with a different profile, since a range of plasma glutamine levels could be found in this group (Fig. 5). This again shows that it is difficult to predict patients that present with deficient plasma glutamine levels by employing single biomarkers alone, due to the high variability within ICU patient groups. It could be postulated that those with the highest CRP values, have more co-morbidities and hence the presence of MOF amongst this group is a reality. The latter situation is regarded as a contra-indication for glutamine supplementation [10, 36]. Again the number of samples analysed in this study may be too small to draw any firm conclusions on the exact CRP cut-off value, and the inclusion of more samples in future studies may provide clear-cut results.

This study had certain limitations. As an observational study, its results reflect only the population included in the study and cannot be extrapolated to all ICU settings. Together with this, calculating the patient’s severity of illness scores are not routinely done in the participating hospitals and could, therefore, not be reported. Body composition measurements were not taken into account in the interpretation of data. Furthermore, as the main aim of the study was to determine plasma glutamine levels on admission to the ICU, glutamine determination was measured only once. A recent study reported that low glutamine levels later during ICU stay correlated with infection rates, length of ICU stay and LOHS [5]. Determination of plasma glutamine during the course of the ICU stay might, therefore, yield better prognostic results and needs to be investigated further.

## Conclusion

This study found glutamine deficiency in 38 % of patients in a mixed ICU. Medical patients had significantly lower plasma glutamine levels, but no gender-related differences could be found. A significant inverse association was found between plasma glutamine and CRP levels, with a cut-off CRP value of 95.5 mg/L above which glutamine levels may become deficient. Whether this can be translated into a clinical recommendation regarding the use of CRP as proxy marker in the determination of the need for glutamine supplementation, is questionable and a larger sample size may be required to better conclude on this. These results, therefore, support earlier findings, which refuted a one-size-fits-all approach with glutamine supplementation. Based on the current literature and the findings of this study, clinicians should consider the holistic profile of the patient, including the patient condition and other biomarkers (e.g., CRP) when selecting patients for glutamine supplementation. This research further contribute baseline measurements for a larger follow up study investigating the plasma glutamine levels of ICU patients at different time points during ICU stay and comparing them to patient outcome.

## Abbreviations

ANCOVA, analysis of covariance; ANOVA, analysis of variance; AUC, area under the curve; CI, confidence interval; CRP, c-reactive protein; EDTA, ethylene diamine tetra-acetic acid; ELISA, enzyme-linked immunosorbent assay; HIV, human immunodeficiency virus; ICU, intensive care unit; IL-6, interleukin-6; LOHS, length of hospital stay; MOF, multiple organ failure; REDOXS, reducing deaths due to oxidative stress; ROC, receiver operating characteristic; SA, South Africa

## References

[1] Dupertuis YM, Meguid MM, Pichard C (2009). Advancing from immunonutrition to a pharmaconutrition: a gigantic challenge. Curr Opin Clin Nutr.

[2] Bergstrom J, Furst P, Noree L, Vinnars E (1974). Intracellular free amino acid concentration in human muscle tissue. J Appl Physiol.

[3] Essen P, Wernerman J, Sonnenfeld T, Thunell S, Vinnars E (1992). Free amino acids in plasma and muscle during 24 hours post‐operatively–a descriptive study. Clin Phys.

[4] Gamrin L, Andersson K, Hultman E, Nilsson E, Essén P, Wernerman J (1997). Longitudinal changes of biochemical parameters in muscle during critical illness. Metabolism.

[5] Pérez-Bárcena J, García-de-Lorenzo A, Buño A, Llompart-Pou JA. A randomized trial of intravenous glutamine supplementation in trauma ICU patients: response to the comments by Ozcelik et al. Intens care med. 2014;40:1397–7. 10.1007/s00134-014-3420-7 25097071

[6] Bollhalder L, Pfeil AM, Tomonaga Y, Schwenkglenks M (2013). A systematic literature review and meta-analysis of randomized clinical trials of parenteral glutamine supplementation. Clin Nutr.

[7] Lin J-J, Chung X-J, Yang C-Y, Lau H-L (2013). A meta-analysis of trials using the intention to treat principle for glutamine supplementation in critically ill patients with burn. Burns.

[8] Yue C, Tian W, Wang W, Huang Q, Zhao R, Zhao Y, Li Q, Li J (2013). The impact of perioperative glutamine-supplemented parenteral nutrition on outcomes of patients undergoing abdominal surgery: a meta-analysis of randomized clinical trials. The Amer surg.

[9] Wischmeyer PE, Dhaliwal R, McCall M, Ziegler TR, Heyland DK (2014). Parenteral glutamine supplementation in critical illness: a systematic review. Crit Care.

[10] Heyland DK, Elke G, Cook D, Berger MM, Wischmeyer PE, Albert M, Muscedere J, Jones G, Day AG. Glutamine and antioxidants in the critically Ill patient a post Hoc analysis of a large-scale randomized trial. JPEN. 2014;0148607114529994.

[11] Rodas PC, Rooyackers O, Hebert C, Norberg A, Wernerman J (2012). Glutamine and glutathione at ICU admission in relation to outcome. Clin Sci.

[12] Andreasen AS, Pedersen-Skovsgaard T, Mortensen OH, Van Hall G, Moseley PL, Pedersen BK (2009). The effect of glutamine infusion on the inflammatory response and HSP70 during human experimental endotoxaemia. Crit Care.

[13] Parry-Billings M, Baigrie RJ, Lamont PM, Morris PJ, Newsholme EA (1992). Effects of major and minor surgery on plasma glutamine and cytokine levels. Arch Surg.

[14] Suliman ME, Qureshi AR, Stenvinkel P, Pecoits-Filho R, Bárány P, Heimbürger O, Anderstam B, Ayala ER, Divino Filho JC, Alvestrand A (2005). Inflammation contributes to low plasma amino acid concentrations in patients with chronic kidney disease. The Am J Clin Nutr.

[15] Oudemans-van Straaten H, Bosman R, Treskes M, Van der Spoel H, Zandstra D (2001). Plasma glutamine depletion and patient outcome in acute ICU admissions. Intens Care Med.

[16] Todd J, Simpson P, Estis J, Torres V, Wub AH (2013). Reference range and short-and long-term biological variation of interleukin (IL)-6, IL-17A and tissue necrosis factor-alpha using high sensitivity assays. Cytokine.

[17] Ledue TB, Rifai N (2003). Preanalytic and analytic sources of variations in C-reactive protein measurement: implications for cardiovascular disease risk assessment. Clin Chem.

[18] Patel VB, Robbins MA, Topol EJ (2001). C-reactive protein: a’golden marker’for inflammation and coronary artery disease. Clev Clin J Med.

[19] Heyland D, Muscedere J, Wischmeyer PE, Cook D, Jones G, Albert M, Elke G, Berger MM, Day AG (2013). A randomized trial of glutamine and antioxidants in critically ill patients. New Engl J Med.

[20] Jackson N, Carroll P, Russell-Jones D, Sönksen P, Treacher D, Umpleby A (1999). The metabolic consequences of critical illness: acute effects on glutamine and protein metabolism. Am J Physiol-Endoc M.

[21] Biolo G, Fleming R, Maggi SP, Nguyen TT, Herndon DN, Wolfe RR (2000). Inhibition of muscle glutamine formation in hypercatabolic patients. Clin Sci.

[22] Karinch AM, Pan M, Lin C-M, Strange R, Souba WW (2001). Glutamine metabolism in sepsis and infection. J Nutr.

[23] Engelen MP, Wouters EF, Deutz NE, Menheere PP, Schols AM (2000). Factors contributing to alterations in skeletal muscle and plasma amino acid profiles in patients with chronic obstructive pulmonary disease. The Am j clin nutr.

[24] Watford M, Chellaraj V, Ismat A, Brown P, Raman P (2002). Hepatic glutamine metabolism. Nutr.

[25] Cynober L, De Bandt J-P (2014). Glutamine in the intensive care unit. Curr Opin Clin Nutr.

[26] Felig P, Wahren J, Karl I, Cerasi E, Luft R, Kipnis DM (1973). Glutamine and glutamate metabolism in normal and diabetic subjects. Diabetes.

[27] Stumvoll M, Perriello G, Nurjhan N, Welle S, Gerich J, Bucci A, Jansson P-A, Dailey G, Bier D, Jenssen T (1996). Glutamine and alanine metabolism in NIDDM. Diabetes.

[28] Planas M, Schwartz S, Arbos M, Farriol M (1993). Plasma glutamine levels in septic patients. JPEN.

[29] Hack V, Schmid D, Breitkreutz R, Stahl-Henning C, Drings P, Kinscherf R, Taut F, Holm E, Dröge W (1997). Cystine levels, cystine flux, and protein catabolism in cancer cachexia, HIV/SIV infection, and senescence. FASEB J.

[30] Van Acker BA, Hulsewé KW, Wagenmakers AJ, Soeters PB, von Meyenfeldt MF (2000). Glutamine appearance rate in plasma is not increased after gastrointestinal surgery in humans. J Nutr.

[31] Viggiano E, Passavanti MB, Pace MC, Sansone P, Spaziano G, Viggiano A, Aurilio C, Monda M, Viggiano A, Pota V (2012). Plasma glutamine decreases immediately after surgery and is related to incisiveness. J Cell Physiol.

[32] Armstrong MD, Stave U (1973). A study of plasma free amino acid levels. III. Variations during growth and aging. Metabolism.

[33] Mihara M, Hashizume M, Yoshida H, Suzuki M, Shiina M (2012). IL-6/IL-6 receptor system and its role in physiological and pathological conditions. Clin Sci.

[34] Sahin H, Mercanligil SM, Inanç N, Ok E (2007). Effects of glutamine-enriched total parenteral nutrition on acute pancreatitis. Eur J Clin Nutr.

[35] Yeh C-N, Lee H-L, Liu Y-Y, Chiang K-C, Hwang T-L, Jan Y-Y, Chen M-F (2008). The role of parenteral glutamine supplement for surgical patient perioperatively: result of a single center, prospective and controlled study. Langenbeck Arch Surg.

[36] Wernerman J (2014). Glutamine supplementation to critically ill patients. Crit Care.

